# Prevalence of Antibiotic Prescription in Patients with Acute Rhinosinusitis Treated by General Practitioners and Otolaryngologists in Germany—A Retrospective Cohort Study

**DOI:** 10.3390/antibiotics11111576

**Published:** 2022-11-09

**Authors:** Claudia B. Bittner, Michael Plach, Hubert Steindl, Dimitri Abramov-Sommariva, Christoph Abels, Karel Kostev

**Affiliations:** 1 Research & Development, Bionorica SE, 92318 Neumarkt in der Oberpfalz, Germany; 2 Epidemiology, IQVIA, 60549 Frankfurt am Main, Germany

**Keywords:** acute rhinosinusitis, antibiotic, general practitioners, otolaryngologist, phytopharmaceutical

## Abstract

(1) Background: The goal of this retrospective cohort study, based on real-world data and conducted in Germany, was to investigate the prevalence of antibiotic (AB) prescription in patients with acute rhinosinusitis (ARS). (2) Methods: Data from the Disease Analyzer database were used for this cross-sectional study. Patients aged ≥18 years diagnosed with acute sinusitis by general practitioners (GPs) and ear, nose, throat (ENT) specialists between January 2012 and December 2020 were included. The main outcome of the study was the proportion of patients with ARS who received an AB prescription on the day of diagnosis or within three days afterwards. The proportion was estimated separately for patients treated by GPs and ENTs, and also for five age groups, as well as women and men. (3) Results: In total, 308,095 patients were diagnosed with ARS (187,838 by GPs and 120,257 by ENTs). 50.9% of patients treated by GPs and 50.0% treated by ENTs received an AB prescription. AB prevalence increased with age from 46.9% in the age group 18–30 years to 55.5% in the age group > 60 years. (4) Conclusions: We have shown a high prevalence of potentially inappropriate AB prescription for adult patients with ARS in both GP and ENT practices and also among both women and men and in several age groups. There is an urgent need for interventions to reduce inappropriate AB use.

## 1. Introduction

Acute sinusitis, also known as acute rhinosinusitis (ARS), is an inflammation of the sinuses with symptoms that last less than 4 weeks (US guidelines) [1] or less than 12 weeks (European guidelines) [2]. ARS is usually caused by viruses including rhinovirus, adenovirus, influenza, and parainfluenza virus, and approximately up to 2% of viral ARS in adults and 5–10% in children develop into bacterial infections [3]. Acute bacterial sinusitis is relatively rare, but may lead to infections of the central nervous system [4].

In a systematic review, Lemiengre et al. showed that 46% of adult patients with uncomplicated ARS were cured after 1 week and 64% after 2 weeks without antibiotic (AB) use. Purulent nasal secretion resolved in 70% of ARS patients treated with AB and 60% of patients taking placebo, i.e., the purulent nasal secretion was stopped by the antibiotic in just 10% of patients. Meanwhile, 13% of people taking antibiotics suffered from side effects triggered by the drug [5].

In the USA, ARS accounts for 20% of all antibiotic prescriptions for adults [3]. Kern and Kostev reported that in Germany 52% of adult patients and 32% of children and adolescents with ARS received AB prescriptions. Furthermore, ARS was the diagnosis associated with the highest odds of receiving AB prescription in pediatric practices [6].

Yet, to the best of our knowledge, no data have been published to date on the prevalence of AB therapy prescriptions to ARS patients in ear, nose, and throat specialist (ENT) practices or on the prevalence of AB prescription in such practices in relation to age, sex, and ARS type.

Therefore, the goal of this retrospective cross-sectional study based on real-world data and conducted in Germany was to investigate the prevalence of AB prescription in patients with ARS.

## 2. Results

In total, 308,095 patients (187,838 by general practitioners (GPs) and 120,257 by ENT) were diagnosed with ARS between January 2012 and December 2020. The age and sex characteristics of the study patients are shown in Table 1. The average age (standard deviation) was 41.1 (15.7) in GP patients and 44.9 (16.6) in ENT patients. The proportion of women was 57.6% in GP patients and 62.3% in ENT patients. Unspecified ARS was the most common diagnosis (55.7%) in GP patients, followed by maxillary (19.5%) and frontal ARS (13.8%). Among ENT patients, maxillary ARS was diagnosed in 49.4% and undefined ARS in 35.7%, while other forms were relatively rare (Table 1).

**Figure 1:**
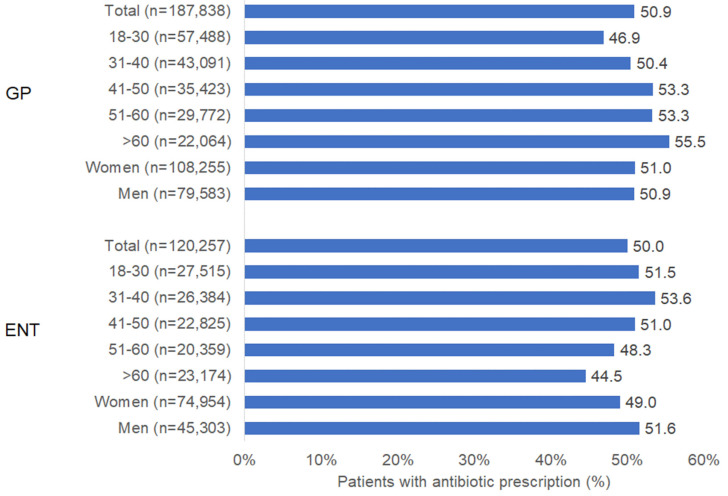
Prevalence of antibiotic prescription in patients with acute sinusitis in GP and ENT practices in Germany.

Of those patients with ARS diagnosis, 50.9% of GP patients and 50.0% of ENT patients received an AB prescription. Figure 1 shows the prevalence of AB prescription stratified by age group and sex. In patients diagnosed by GPs, AB prescription prevalence increased with age from 46.9% in the age group 18–30 years to 55.5% in the age group > 60 years. However, the reverse trend was observed in ENT practices, with the highest prevalence (53.6%) in the age group 31–40 years and the lowest (44.5%) in the age group > 60 years. There was only a small difference in AB prevalence between female and male patients (Figure 1).

## 3. Discussion

In this large retrospective cohort study including 308,095 patients with ARS, the prevalence of antibiotic prescription was around 50% without any clinically relevant difference between patients attending GPs and those treated by ENT specialists. The unnecessary prescription of AB therapy continues to pose a major challenge in daily clinical practice. This is because the clinical symptoms of viral and bacterial ARS are similar and several methods including endoscopic investigations and laboratory tests are needed to correctly diagnose bacterial ARS [7].

The trends found in our study are in line with those observed in other published studies from Spain [8], the United Kingdom [9], Canada [10], and the USA [11]. In Spain, 62% of patients with viral and 76% with post-viral ARS received antibiotics [8]. In English primary care, 82% of patients with ARS received antibiotic prescriptions. Interestingly, however, only 11% of these prescriptions were identified as appropriate by experts [9]. In Canada, 68% of patients aged 19–64 and 62% aged >64 received AB for ARS. The fact that AB were only needed in 18% of patients in both age groups in this study once again shows the high rate of unnecessary antibiotic prescription [10]. In the study conducted in the USA, 81% of study patients with ARS were treated with antibiotics [11].

On the other hand, the European Position Paper on Rhinosinusitis and Nasal Polyps (EPOS) [2], the German Society of Oto-Rhino-Laryngology, Head and Neck Surgery, and the German College of General Practitioners and Family Physicians [12] recommend avoiding AB when there is no indication for their use.

Criteria for the appropriate prescription of antibiotics include persistent symptoms over at least 10 days, severe symptoms, and worsening symptoms. Unfortunately, there is often no difference in the proportion of antibiotic prescription between patients who meet the criteria for the prescription of antibiotics and those who do not [13].

The main negative consequence of inappropriate antibiotic use is the development of antimicrobial resistance, which has been a significant global public health challenge for many years and is the cause of severe infections and increased mortality [14,15]. In addition, antibiotics negatively affect the gut microbiota, which may promote the development and aggravation of disease [16].

In recent years, a number of systematic reviews evaluating the effectiveness of medication in the treatment of ARS have been performed and summarized in the European Position Paper on Rhinosinusitis and Nasal Polyps [2]. For example, paracetamol may help to relieve nasal obstruction and rhinorrhea. Antihistamines alone have a limited beneficial effect on the severity of overall symptoms in adults with ARS, but only on the first and second day of treatment. Decongestants alone may have a slight positive effect on subjective measures; the current evidence suggests that multiple doses of nasal decongestants may have a slight positive effect on subjective assessments of nasal congestion in adults with the common cold. However, it has been suggested that combinations of antihistamines, analgesics, and decongestants have some general benefit in adults with ARS [2].

Treatment with herbal medicinal products, such as a patented extract of five herbal drugs (gentian root, primula flower, sorrel herb, elder flower, and verbena herb) (BNO 1016) or defined eucalyptus extracts can be recommended as an alternative to antibiotics for the treatment of ARS [2,12]. Herbal medicinal products are sustainable and help to preserve the microbiota when used [17]. In a large retrospective study involving more than 100,000 patients, Martin et al. showed that the use of different phytopharmaceuticals for acute respiratory infections including ARS was associated with a significantly reduced need for antibiotic prescriptions in the further course of the disease and also with significantly shorter sick leaves [18].

In their investigation, performed in the USA, Milani et al. demonstrated that primary care physician and patient education followed by regular feedback could significantly reduce the frequency of inappropriate antibiotic prescribing for acute respiratory tract infections [19].

A further way to avoid the inappropriate prescription of AB is the use of point-of-care tests which could not only reduce antibiotic use but also patient pressure for antibiotic prescriptions [20]. The use of delayed AB prescribing can also be implemented to this end [21].

Limitations of the present study include the lack of information on ARS severity, patients’ preference for AB, and patients’ sociocultural variables. However, our study has several strengths including a very large sample size, use of data from clinical practice as well as use of data from both GP and ENT practices.

In conclusion, we have shown a high prevalence of potentially inappropriate AB prescription for adult patients with ARS in both GP and ENT practices, in both women and men, and in several age groups. There is an urgent need for interventions to reduce inappropriate AB use.

## 4. Materials and Methods

### 4.1. Database

Data from the Disease Analyzer database (IQVIA) were used for this cross-sectional study. This database has been extensively described in the literature [22]. Briefly, the Disease Analyzer database contains demographic, diagnosis, and prescription data obtained from general and specialized practices in Germany in an anonymized format. The sampling method for this database is based on summary statistics from all doctors in Germany published yearly by the German Medical Association. The panel design of the database is determined using several strata (i.e., age of physician, specialist group, community size category, and German federal state). Finally, previous research has shown that this database is representative of private practices in Germany [22].

### 4.2. Study Population and Outcome

Patients aged ≥ 18 years diagnosed with acute sinusitis (ICD-10: J01) by general practitioners and ENT specialists between January 2012 and December 2020 were included. Patients with a diagnosis of acute or chronic sinusitis prior to the index date and patients with prescriptions of AB, nasal sprays with or without corticosteroid, analgesics (paracetamol or ibuprofen), or herbal medications for ARS therapy within 90 days prior to the index date were excluded.

The main outcome of the study was the proportion of patients with ARS who received an AB prescription on the day of diagnosis or within three days afterwards. The proportion was estimated separately for patients treated by GPs and ENT specialists, patients in five age groups (18–30, 31–40, 41–50, 51–60, >60), and women and men.

This study is of a descriptive nature, and no hypotheses were tested. Analyses were performed using SAS 9.4 (SAS Institute, Cary, NC, USA).

## Figures and Tables

**Table 1:** Baseline characteristics of study patients.

Variable	GP (*n*, %)	ENT (*n*, %)
Total	187,838	120,257
Age (mean, SD)	41.1 (15.7)	44.9 (16.6)
18–30	57,488 (30.6%)	27,515 (22.9%)
31–40	43,091 (22.9%)	26,384 (21.9%)
41–50	35,423 (18.9%)	22,825 (19.0%)
51–60	29,772 (15.8%)	20,359 (16.9%)
>60	22,064 (11.7%)	23,174 (19.3%)
Female	108,255 (57.6%)	74,954 (62.3%)
Male	79,583 (42.4%)	45,303 (37.7%)
**ARS Type**		
Maxillary sinusitis	36,618 (19.5%)	59,416 (49.4%)
Frontal sinusitis	25,868 (13.8%)	6328 (5.3%)
Ethmoidal sinusitis	1319 (0.7%)	4061 (3.4%)
Pansinusitis	9728 (5.2%)	5193 (4.3%)
Other ARS	9541 (5.1%)	2363 (2.0%)
Unspecified ARS	104,692 (55.7%)	42,752 (35.7%)

## Data Availability

The data presented in this study are available on request from the corresponding author.
